# Dexmedetomidine Attenuates Neuroinflammatory–Induced Apoptosis after Traumatic Brain Injury via Nrf2 signaling pathway

**DOI:** 10.1002/acn3.50878

**Published:** 2019-09-03

**Authors:** Fayin Li, Xiaodong Wang, Zhijie Zhang, Xianlong Zhang, Pengfei Gao

**Affiliations:** ^1^ Department of Anesthesiology The Affiliated Huaian No. 1 People's Hospital of Nanjing Medical University 6 Beijing Road West Huaian 223002 Jiangsu China; ^2^ Department of Neurosurgery The Affiliated Huaian No. 1 People's Hospital of Nanjing Medical University 6 Beijing Road West Huaian 223002 Jiangsu China

## Abstract

**Objective:**

Dexmedetomidine (DEX) exhibits neuroprotective effects as a multifunctional neuroprotective agent in numerous neurological disorders. However, in traumatic brain injury (TBI), the molecular mechanisms of these neuroprotective effects remain unclear. The present study investigated whether DEX, which has been reported to exert protective effects against TBI, could attenuate neuroinflammatory‐induced apoptosis and clarified the underlying mechanisms.

**Methods:**

A weight‐drop model was established, and DEX was intraperitoneally injected 30 min after inducing TBI in rats. The water content in the brain tissue was measured. Terminal deoxynucleotidyl transferase‐mediated dUTP nick‐end labeling (TUNEL) assays were performed on histopathological tissue sections to evaluate neuronal apoptosis. Enzyme‐linked immunosorbent assay and PCR were applied to detect the levels of the inflammatory factors, TNF‐*α*, IL‐1*β*, IL‐6, and NF‐*κ*B.

**Results:**

TBI–challenged rats exhibited significant neuronal apoptosis, which was characterized via the wet‐to‐dry weight ratio, neurobehavioral functions, TUNEL assay results and the levels of cleaved caspase‐3, Bax upregulation and Bcl‐2, which were attenuated by DEX. Western blot, immunohistochemistry, and PCR results revealed that DEX promoted Nrf2 expression and upregulated expression of the Nrf2 downstream factors, HO‐1 and NQO‐1. Furthermore, DEX treatment markedly prevented the downregulation of inflammatory response factors, TNF‐*α*, IL‐1*β* and NF‐*κ*B, and IL‐6.

**Interpretation:**

Administering DEX attenuated inflammation‐induced brain injury in a TBI model, potentially via the Nrf2 signaling pathway.

## Introduction

The pathophysiological mechanisms of traumatic brain injury (TBI) include primary brain tissue disruption and secondary brain injury, including excitotoxic damage, inflammation, increased vascular permeability, and oxidative stress.^1^, ^2^ Among these events, inflammation is considered an important contributor to the TBI pathophysiology and exacerbates neuronal damage during the process of secondary insult, which is characterized by peripheral inflammation, cell infiltration, and secretion of inflammatory mediators.^3^, ^4^ Sustained and excessive inflammation can exacerbate subsequent neurological impairment through secretion of proinflammatory mediators such as interleukin (IL)‐1*β*, tumor necrosis factor (TNF)‐*α*, and IL‐6.^1^ Previous studies have confirmed that these acute‐phase cytokines are regulated via the nuclear factor kappa B (NF‐*κ*B) signaling pathway after TBI and that NF‐*κ*B expression increases rapidly after brain injury.^5^

Inflammation is linked with oxidative stress, or elevated intracellular levels of reactive oxygen species (ROS), which are considered the most potent inflammatory mediators.^6^ Nuclear factor erythroid 2‐related factor 2 (Nrf2) is an important transcription factor that regulates cellular antioxidant activity and has been shown to protect organs in several animal models.^7^, ^8^ Nrf2–mediated antioxidant gene expression can reduce the macrophage M1 phenotype and ROS production; therefore, activating the Nrf2 pathway may inhibit the progression of inflammation and may be a potential therapeutic strategy for many disorders such as cardiovascular diseases, neurodegenerative diseases and multiple sclerosis.^9^, ^10^ Moreover, Nrf2 has been reported to be an important regulator in protecting against TBI–induced secondary brain injury. Nrf2 activation may also be involved in the neuroprotective effect of DEX after TBI.

Dexmedetomidine (DEX) is a central adrenoceptor *α*2‐adrenergic receptor agonist with sedative, analgesic, and anti‐stress reaction effects.^11^, ^12^ DEX affects neurons and exerts protective effects by decreasing catecholamine and glutamate levels and preventing neuronal apoptosis.^13^ Previous studies demonstrated that suppressing proinflammatory cytokine production in the brain played a crucial role in DEX–induced neuroprotection after cerebral ischemia/reperfusion injury.^14^, ^15^ However, the effects of DEX on the acute inflammatory responses after TBI and the exact mechanisms of these effects remain unclear.

In the present study, we examined the mechanisms by which DEX protects against acute inflammatory responses and cellular apoptosis and maintains neurological function during the acute phase of TBI. We further assessed the pathway of DEX relative to TBI–induced neuroinflammation.

## Materials and Methods

### Animals and TBI model

All experimental protocols were approved by the Animal Care and Use Committee of Fujian Medical University in accordance with the rules of the National Institutes of Health (NIH). The TBI model was established as described in a previous study.^16^ Male Sprague‐Dawley rats weighing 250–280 g were purchased from the Experimental Animal Center of Nanjing Medical University. Rats were anesthetized intraperitoneally (i.p.) with sodium pentobarbital (30 mg/kg) after being housed at a controlled temperature (25 ± 2°C) with free access to food and water for 7 days. The rats were randomly divided into five groups: Sham, Sham + DEX, TBI, TBI + vehicle, and TBI + DEX. All rats were placed in a stereotaxic frame, and their skulls were exposed through a 2‐cm midline incision through the skin on the scalp. A 6‐mm hole was made over the left parietal cortex. TBI was induced by dropping a 450‐g weight from a height of 1.5 cm, and then the scalp wound was sutured. The Sham groups underwent this procedure but without the weight drop.

### Experimental protocol

Rats in the Sham + DEX and TBI + DEX groups were injected intraperitoneally with DEX (Xincheng, Jiangsu, China) at a dose of 25 *μ*g/kg dissolved in saline as a 1 *μ*g/μL solution. Rats in the TBI + vehicle group were simultaneously administered equal volumes of the vehicle solution. Rats in the TBI + DEX and TBI + vehicle groups were administered equal volumes of saline solution 30 min after TBI. The drug doses used in this study were the same as those used in a previous study.^16^

### Measurement of the brain water content

Brain water content was measured as described in a previous report.^17^ Animals were sacrificed, and the left brain cortical tissue was collected. The tissue was positioned directly over the injury site, covering the contusion and the penumbra. The fresh tissue was weighed to record the wet weight, dried for 72 h at 80°C, then weighed to record the dry weight. The brain water content was calculated using the formula: [(wet weight − dry weight)/wet weight] × 100%.

### Neurobehavioral assessments

The severity of the neurological deficits was evaluated at 24 h posttreatment using the neurological severity score (NSS) system, which uses a scale of 0–18 (0 = minimum deficit and 18 = maximum deficit).^18^ The NSS was assessed based on the motor, sensory, reflexive, and balance functions. Higher scores were associated with greater injuries. All animals were measured 1, 3, 7, and 14 days after TBI. Two independent investigators who were blinded to the experimental groups conducted the behavioral tests.

### Brain tissue preparation

For the immunohistochemical staining and TUNEL analysis, the rats were sacrificed and perfused with normal saline, then fixed with 500 mL of 4% paraformaldehyde 24 h after TBI. The brains were quickly removed from the skulls and fixed with 4% paraformaldehyde. The brains were then dehydrated in 15% and 30% sucrose until completely infiltrated. For the quantitative real‐time PCR, ELISA and Western blot analyses, the rats were infused with normal saline only, then the brain tissues were quickly removed, and the pericontusive cortexes were collected and stored at −80°C until use.

### Enzyme‐linked immunosorbent assay (ELISA)

The cortexes were collected and homogenized with lysis buffer. The lysate was centrifuged at 12,000*g* for 20 min at 4°C. The concentrations of TNF‐*α*, IL‐1*β*, and IL‐6 were detected using ELISA kits ( Boster Bio‐Engineering Ltd. Co, Wuhan, China).The OD values of the samples were detected using a microplate reader (MULTISKAN MK3, Thermo).

### Real‐time quantitative polymerase chain reaction (RT‐qPCR)

Total RNA was extracted from the injured foci of the cortex tissues using RNAiso Plus (TaKaRa Bio, Dalian, China). The isolated RNA was reverse transcribed to cDNA using the Prime Script RT reagent kit to avoid RNA degradation. The primer sequences were designed as follows:

IL‐1*β*: F, 5′‐CAG CAA TGG TCG GGA CAT AGTT‐ 3′; R, 5′‐ GCA TTA GGA ATA GTG CAG CCA TCT‐3′;

NF‐ *κ*B: F, 5′‐ TGG GGA CCA GGA AGA GGT GGC‐ 3′; R, 5′‐ GCT GTG GCC CTG ACA GTA GCC ‐ 3′;

IL‐6: F, 5′‐CCA ACT TCC AAT GCT CTC CTA ATG‐3′; R, 5′‐ TTC AAG TGC TTT CAA GAG TTG GAT‐3′;

TNF‐*α*: F, 5′‐GGC TGC CTT GGT TCA GAT GT‐3′; R, 5′‐CAG GTG GGA GCA ACC TAC AGTT ‐‐3′;

NQO1: F: 5′‐CAT TCT GAA AGG CTG GTT TGA‐3′; R:5′‐CTA GCT TTG ATC TGG TTG TCAG‐3′;

HO‐1: F: 5′‐ATC GTG CTC GCA TGA ACA CT‐3′; R: 5′‐CCA ACA CTG CAT TTA CAT GGC‐3′;

*β*‐actin: F, 5′‐AGT GTG ACG TTG ACA TCC GTA‐3′; R: 5′‐GCC AGA GCA GTA ATC TCC TTCT‐3′. *β*‐ actin was used as the internal reference.

### Western blot analysis

Protein was extracted, diluted in sodium dodecyl sulfate loading buffer, electrophoresed on 10 or 15% sodium dodecyl sulfate‐polyacrylamide gels, and transferred to polyvinylidene fluoride membranes (Bio‐Rad Lab, Hercules, CA). The membranes were blocked with 5% nonfat milk for 2 h, then incubated overnight at 4°C with Nrf2 (1:1000, Abcam, Cambridge, MA), NF‐*κ*B (Cell Signaling Technology, Beverly, MA), Bcl‐2 (1:200, Santa Cruz, CA), Cleaved caspase‐3 (1:1000, Cell Signaling, Danvers, MA), Bax (1:200, Santa Cruz, CA), NF‐*κ*B (1:1000, Cell Signaling, Danvers, MA), uncleaved caspase‐3 (1:400, Cell Signaling, Danvers, MA), HO‐1 (1:200, Santa Cruz Biotechnology), NQO‐1 (1:1000, Abcam, Cambridge, MA) and *β*‐actin (1:5000, Bioworld Technology, MN). After washing three times with TBST (15 min each), the membranes were incubated with the secondary antibody (1:1000, Bioworld Technology, MN) at room temperature for 2 h. The protein bands were exposed to the Tanon‐5200 Chemiluminescent Imaging System and quantified using the associated software (Bio‐Rad Laboratories, Inc.).

### Immunohistochemical staining

The 5‐*μ*m paraffin‐embedded sections were rehydrated and microwaved for antigen retrieval with EDTA and pretreated with 0.3% H_2_O_2_. Sections were washed for 15 min in phosphate‐buffered saline (PBS), then immersed in 10% goat serum containing PBS‐Triton (0.3%) for one hour at room temperature. The sections were then incubated overnight at 4°C with a primary antibody against Nrf2 (1:400, Santa Cruz, CA). The sections were then washed three times in PBS and counterstained with diaminobenzidine and hematoxylin. Control tissues were similarly stained without the primary antibody step.

### Terminal deoxynucleotidyl transferase‐mediated dUTP nick‐end labeling (TUNEL) analysis

The apoptotic cells were detected using a TUNEL detection kit (Roche Inc., Indianapolis, IN). The sections were incubated at 37°C with the TUNEL reaction fluid for 60 min, then digoxigenin‐conjugated dUTP was added to the 3′‐OH ends of the fragmented DNA. The sections were then washed three times with PBS for 45 min followed by blocking with 10% goat serum in 0.1 mol/L Tris for 15 min. The slides were washed three more times, then coverslipped with mounting medium. DNA was fixed with streptavidin‐ horseradish peroxidase (1:40 dilution) and counterstained with the DAB chromogen. Atrophied apoptotic cells with crimped brown nuclei were visualized under a ZEISS HB050 inverted microscope. Two observers blinded to the experiment assessed the positive cells. TUNEL–positive cells in four regions of the slide were quantified. The average percentage of these regions was calculated and included in the final data.

### Statistical analysis

All statistical analyses were performed using SPSS, version 17.0 (SPSS, Inc., Chicago). Data are expressed as the mean ± SD, and the differences were analyzed using ANOVA and Tukey’s tests. Statistical significance was set at *P* < 0.05.

## Results

### DEX treatment reduced TBI in rats

**Figure 1:**
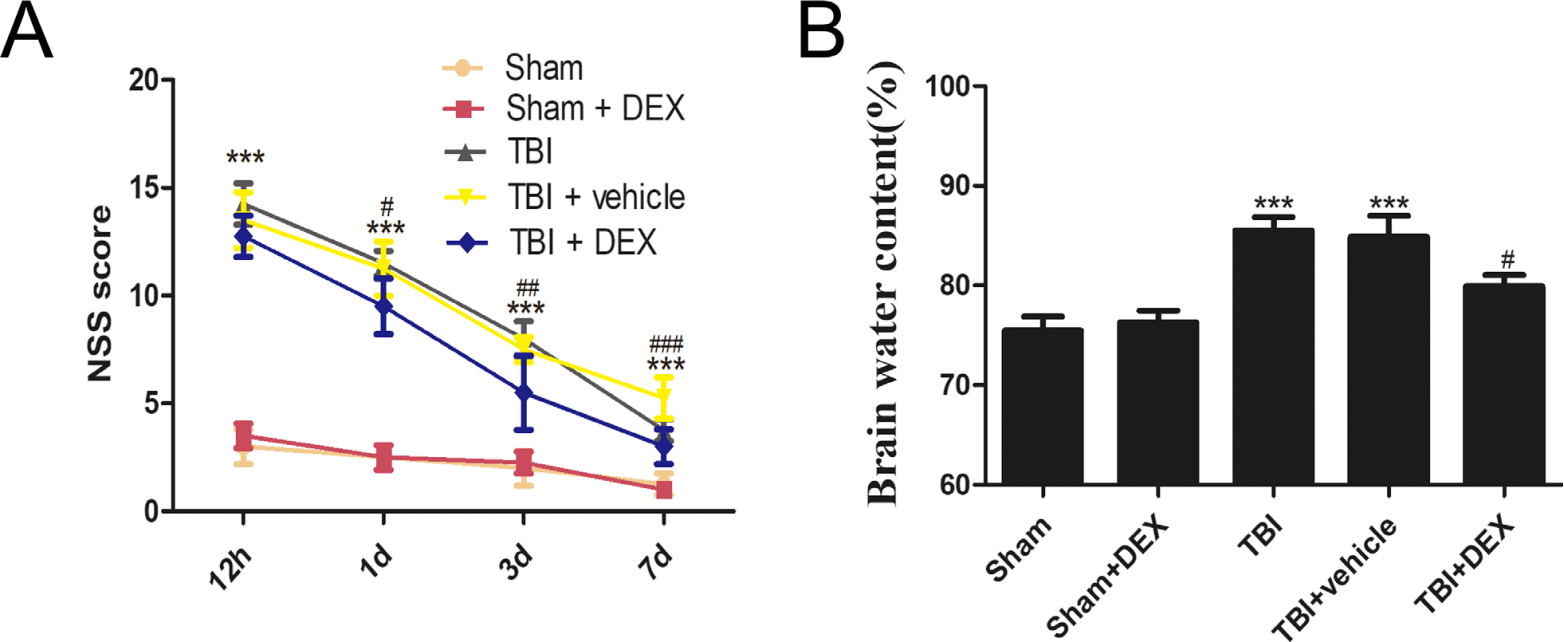
DEX protects rats against secondary brain injury after TBI (*n* = 6). DEX administration improves neurobehavior after TBI (A). Rats subjected to TBI or treated with vehicle had increased brain water content as compared with sham group. Brain water content was significant lower in the groups with administration of DEX than vehicle treated group (B). Data are expressed as the mean ± SEM. ***P < 0.001, versus sham group; ^#^ *P* < 0.05, ^##^ *P* < 0.05 and ^###^ *P* < 0.001 versus TBI + vehicle group.

NSS scores were evaluated at 12 h and 1, 3 and 7 days post‐TBI (Fig. 1A). NSS scores were higher in the TBI and TBI + vehicle groups than in the Sham and Sham + DEX groups 3 days after inducing TBI (*P* < 0.001). However, at 1, 3 and 7 days post‐TBI, the NSS scores declined in the TBI + DEX group compared with those in the TBI and TBI + vehicle groups. No significant differences were observed between 12 h and 7 days post‐TBI. All rats were sacrificed 1 day after TBI for analyses in subsequent experiments. The brain water content was evaluated 24 h post‐TBI (Fig. 1B). Cerebral edema due to TBI and TBI + vehicle was significantly increased compared with that in the Sham groups 24 h after TBI. No significant differences were observed between the TBI and TBI + vehicle groups.

**Figure 2:**
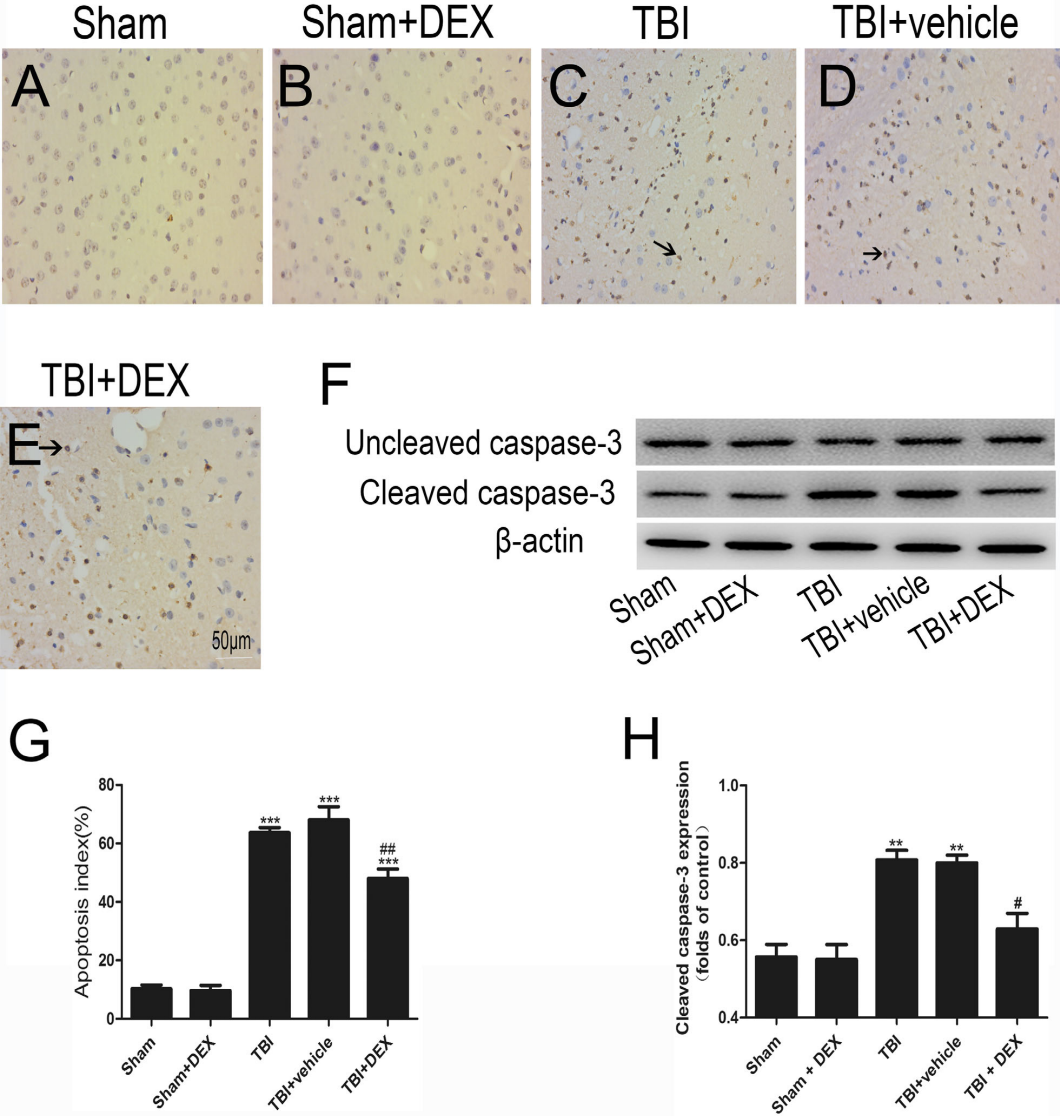
The apoptotic index was determined using TUNEL assays after TBI (n = 6). DEX treatment significantly decreased the percentage of apoptotic cells（A–E, G） and cleaved caspase‐3 expression after TBI（F,H）. Data are presented as the mean ± SEM; **P < 0.01, ***P < 0.001 vs sham group; #*P* < 0.05, ##P *<* 0.01 vs TBI + vehicle group. Black arrows: Positive neuronal cell.

Histopathological alterations in the cortex were investigated via TUNEL assay. The Sham group appeared to have a normal structure. The sham group contained few positive cells (Fig. 2). Furthermore, the apoptotic index increased post‐TBI. The DEX‐treated‐ and untreated‐TBI groups did not differ, and fewer apoptotic cells were found in the TBI + DEX group (*P* < 0.05).

Caspase‐3 was expressed around the cortex, and the protein expression was elevated 24 h after TBI. Cleaved caspase‐3 expression was significantly increased in the TBI and TBI + vehicle groups compared with that in the Sham and Sham + DEX groups and was notably suppressed after administering DEX (*P* < 0.05).

### DEX reduced apoptotic factor expression

**Figure 3:**
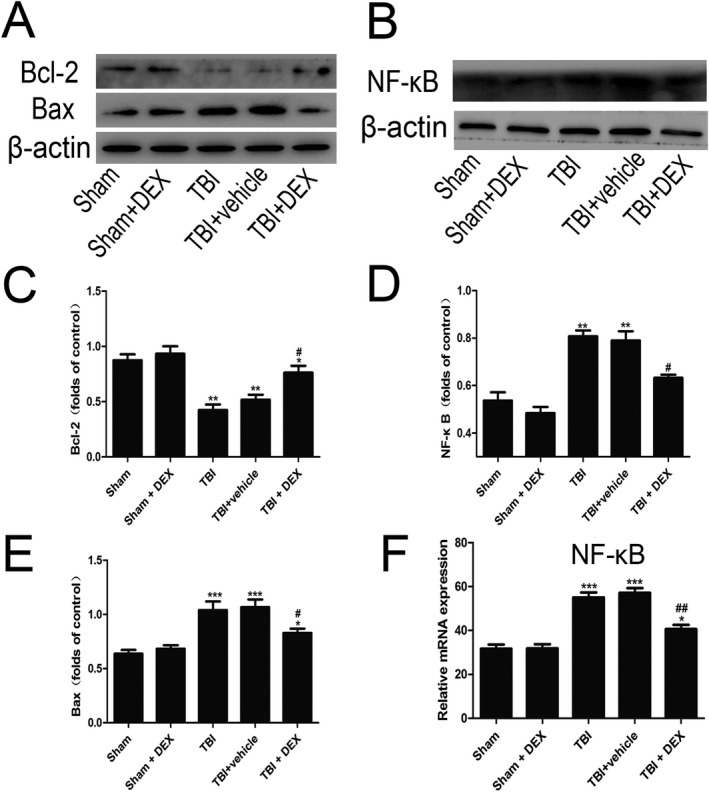
The effect of DEX on Bax, Bcl‐2 (A, C and E) and NF‐κB expression (B, D and F) in cortical neuronal cells in the rat TBI model was assessed with Western blot analysis and RT‐qPCR (n = 6). Data are presented as the mean ± SEM. *P < 0.05, **P < 0.01, ***P < 0.001 vs. sham group; #*P* < 0.05, ##*P* < 0.01 vs. TBI + vehicle group.

The protective effects of DEX against TBI–induced neuronal apoptosis were examined via Western blot analysis (Fig. 3). Expression of the proapoptotic factor, Bax, increased following TBI compared with that of the Sham group (Fig. 3A), whereas expression of the antiapoptotic factor, Bcl‐2, decreased compared with that of the Sham group (Fig. 3A). These effects were reversed in the TBI–induced rats treated with DEX.

### DEX decreased proinflammatory cytokine expression following TBI

The expressions of TNF‐*α*, IL‐1*β* and IL‐6 in the pericontusive tissues were detected via RT‐qPCR and ELISA at 24 h after TBI. TNF‐*α*, IL‐1*β* and IL‐6 were expressed at low levels in the Sham group. RT‐qPCR and ELISA analysis confirmed that TNF‐*α*, IL‐1*β* and IL‐6 were upregulated after TBI, while DEX treatment significantly reduced the proinflammatory cytokine expression.

**Figure 4:**
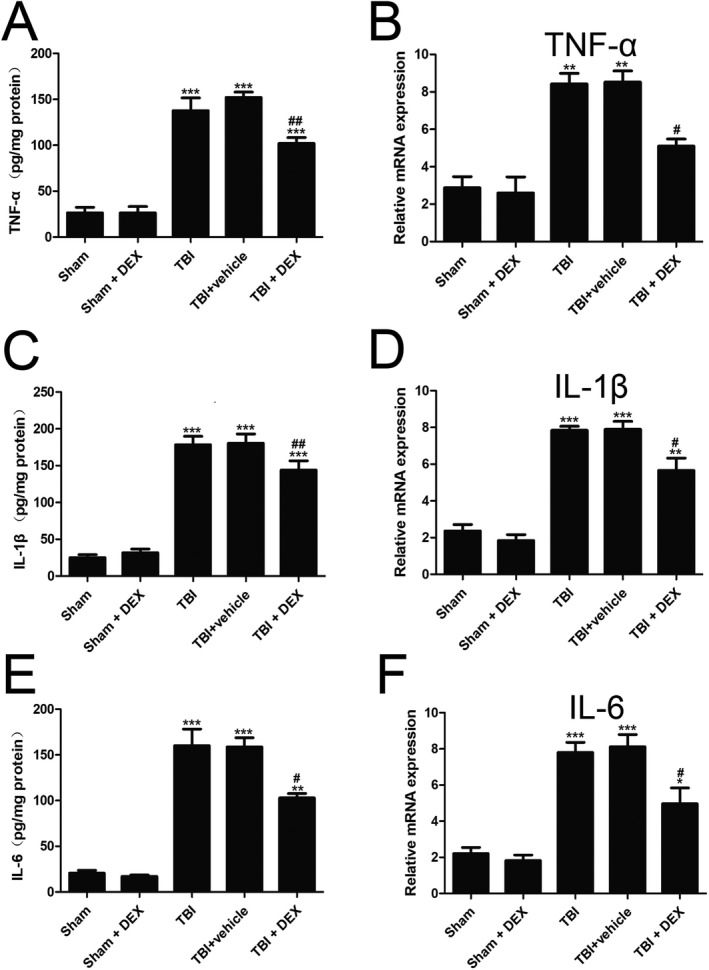
DEX treatment decreased the expression levels of pro‐inflammatory cytokines after TBI (n = 6). The mRNA levels of TNF‐α, IL‐1β, and IL‐6 in peri‐contusive cortex were determined by RT‐qPCR after TBI (A, C and E). And, the protein levels were measured by ELISA post‐TBI (B, D and F). Data are presented as the mean ± SEM. *P < 0.05, **P < 0.01, ***P< 0.001 vs. sham group; #*P* < 0.05, ##*P* < 0.01 vs. TBI + vehicle group

To further investigate whether the neuroprotective effect of DEX was due to NF‐*κ*B inhibition, RT‐qPCR and Western blot assay were performed postinjury. TBI significantly increased the NF‐*κ*B expression compared with that in the Sham group (Fig. 4A). In contrast, DEX treatment markedly decreased the NF‐*κ*B expression in the pericontusional cortex postinjury compared with that in the TBI + vehicle group (*P* < 0.05).

### DEX promoted Nrf2 protein and downstream factor expression

Western blot analysis and immunohistochemistry were applied to detect Nrf2 distribution and expression levels. Nrf2 was mainly located in the cytoplasm in the Sham and Sham + DEX groups (Fig. 4A and 4), while Nrf2 expression was increased in the TBI and TBI + vehicle groups. Administering DEX significantly increased the Nrf2 levels (*P* < 0.05).

**Figure 5:**
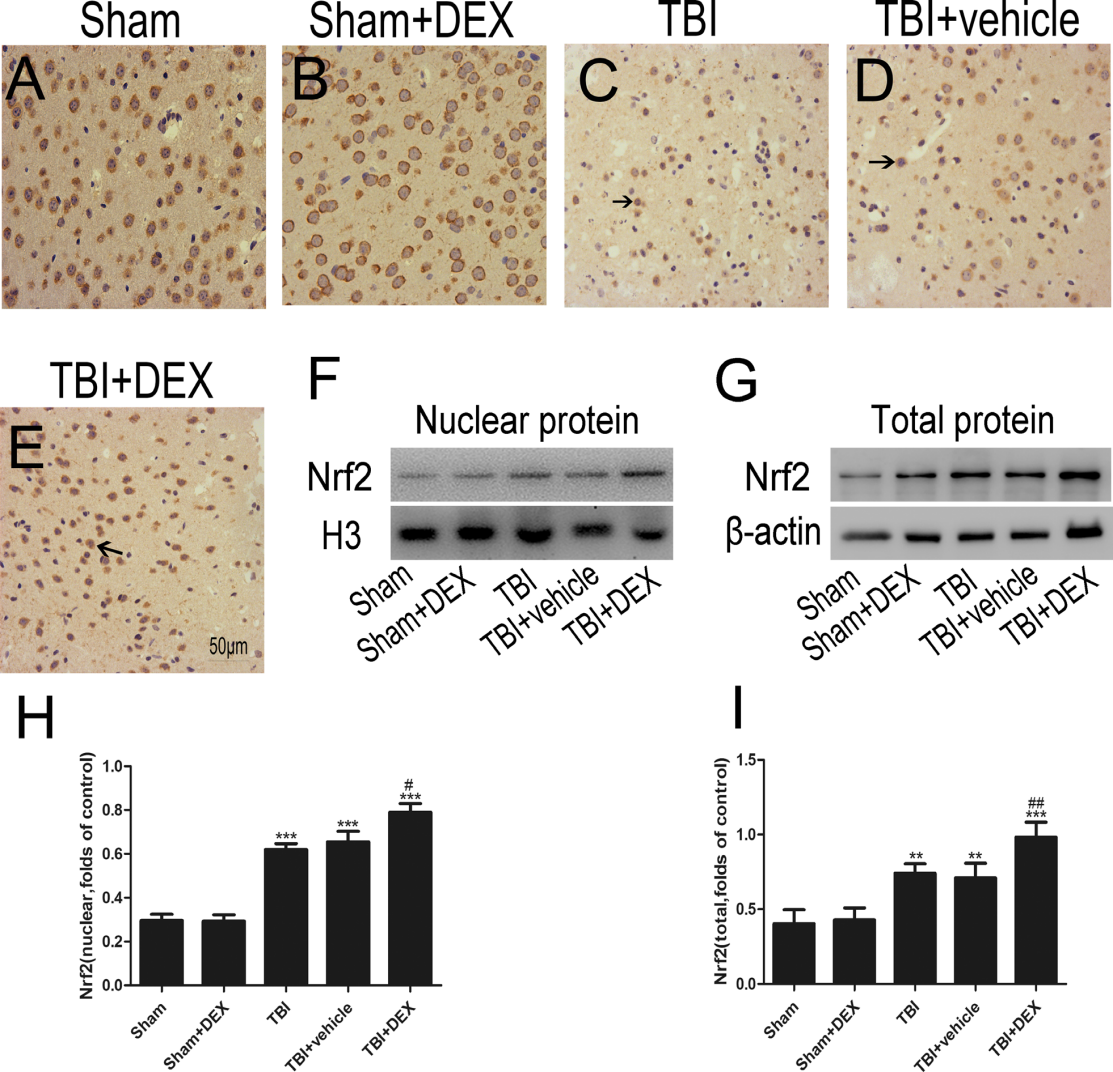
DEX promotes translocation of Nrf2 from the cytoplasm to the nucleus and enhances Nrf2 binding. (A–E) The representative photomicrographs showing Nrf2 immunohistochemistry of brain tissue from the different groups after TBI. (F–I) Total and nuclear Nrf2 expression after DEX treatment following TBI were measured using western blot (*n* = 6). Bars represent the mean ± SEM. **P < 0.01, ***P < 0.001 compared with the sham group; #*P* < 0.05, ##*P* < 0.01 versus TBI + vehicle group. Black arrows: Nrf2 positive neuronal cell.

NQO‐1 and HO‐1 protein expression was enhanced significantly following TBI (Fig. 5; *P* < 0.05). DEX treatment further enhanced protein levels compared with those of the TBI + vehicle group. These results demonstrated that DEX induced Nrf2 downstream factor expression (Fig. 5A and 5; *P* < 0.05).

## Discussion

**Figure 6:**
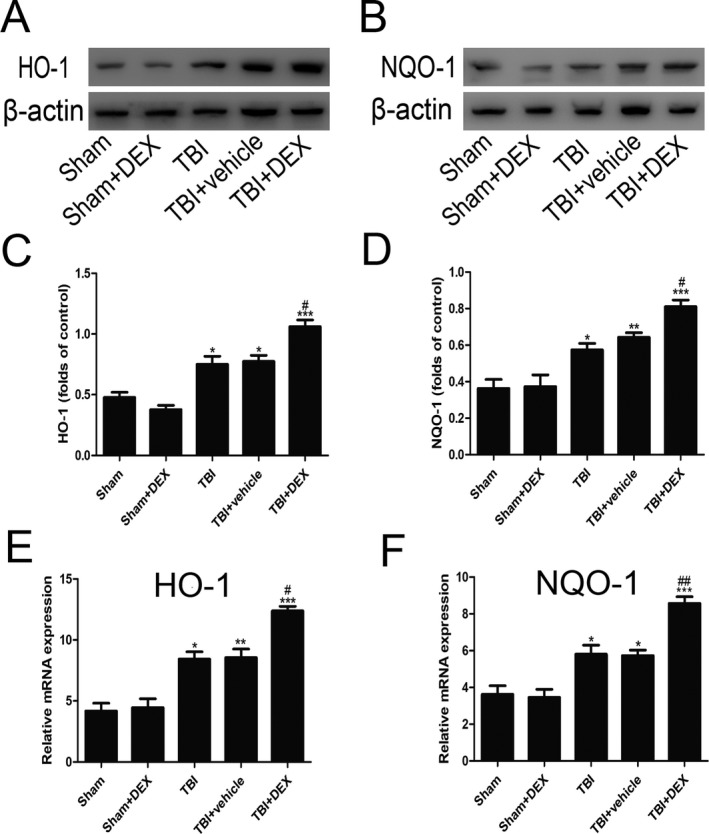
DEX upregulates the expression of HO‐1 (A, C and E) and NQO‐1 (B, D and F) on both protein and mRNA levels (n = 6). β‐actin was used as a loading control. Data are presented as the mean ± SEM; *P < 0.05, **P < 0.01 and ***P < 0.001 vs sham group; #*P* < 0.05, ##*P* < 0.01 vs TB1 + vehicle group. DEX upregulates the expression of Nrf2 downstream factors on both protein and mRNA levels (*n* = 6). *β*‐actin was used as a loading control. Data are presented as the mean ± SEM; *P < 0.05, **P < 0.01 and ***P < 0.001 versus sham group; #*P* < 0.05, ##*P* < 0.01 versus TB1 + vehicle group.

Inflammatory response is associated with proinflammatory cytokine production and plays a critical role in the progression of secondary damage.^19^ Brain inflammatory reactions must be mitigated to decrease morbidity and mortality.^16^ The present study revealed significant neuronal apoptosis following TBI, as evidenced by changes in the TUNEL analysis, neurobehavioral tests and increased cleaved caspase‐3, which is consistent with the findings of previous studies. Bcl‐2 family proteins regulate neuronal apoptosis. The Bcl‐2 family consists of antiapoptotic (Bcl‐2) and proapoptotic (Bax) factors. The pro‐apoptotic family members trigger cytokine release into the cytoplasm, leading to caspase activation.^13^, ^20^, ^21^ In present study, Bax and Bcl‐2 expression levels were detected using Western blotting. The results suggested that TBI induced Bax upregulation and Bcl‐2 downregulation, which were improved after administering DEX (Fig. 6).

DEX presynaptically inhibits noradrenaline release and exerts various effects, including sedation and neuroprotection, although its neuroprotective mechanism remains unclear.^22^ In the present study, a rat model was established to investigate whether DEX could alleviate neuroinflammation‐induced apoptosis after TBI. TBIinduced inflammation in the post‐TBI cerebral cortex by overexpressing TNF‐*α*, IL‐1*β*, and IL‐6, while DEX treatment alleviated this inflammation. NF‐*κ*B is a proinflammatory regulatory cytokine that can intensify inflammatory responses.^23^ Upregulation of Nrf2 activity could improve clinical outcomes through reduction of oxidative stress and post‐traumatic inflammatory responses.^24^ By contrast, repression of the NF‐*κ*B inhibitor system in an experimental model of closed‐head injury promoted neuronal cell death, worsened the neurological outcome and increased post‐traumatic mortality rate. Previous study proved found that NF‐*κ*B activation was prolonged in glial cells and neurons following TBI.^25^ In the present study, DEX treatment effectively reduced NF‐*κ*B expression, suggesting that DEX may attenuate post‐traumatic inflammatory responses by modulating NF‐*κ*B activation. This finding is consistent with the cytoprotective mechanisms of action associated with Nrf2, which promote Bcl‐2 protein expression and Bax downregulation. Other proapoptotic factors promote NF‐*κ*B activity and are involved in cell death and tissue damage.

Nrf2 is an important protective protein and plays a key role in cells adapting to oxidative stress by elevating phase *α* enzymes, such as NQO1 and HO‐1, which are activated after TBI.^26^, ^27^, ^28^ Noxious stimulation can increase Nrf2 expression and its translocation into the nucleus to bind with antioxidant response‐element sequences.^10^, ^29^ To confirm that the Nrf2 signaling pathway is involved in the neuroprotection by DEX, Nrf2 protein expression in the brain tissue of rats with TBI was assessed after administering DEX. Downstream factors of the Nrf2 pathway, including NQO‐1 and HO‐1, were investigated. The present study demonstrated that Nrf2, NQO1, and HO‐1 protein expressions were increased significantly after administering DEX. Furthermore, DEX promoted Nrf2 migration from the cytoplasm to the nucleus post‐TBI. Total Nrf2 protein was significantly upregulated after TBI and was even higher after administering DEX. These findings illustrated the influence of DEX on Nrf2 protein expression and translocation and demonstrated that DEX reduced neuronal apoptosis and improved Nrf2 expression. Thus, Nrf2 signaling is an important mechanism underlying the positive effects of DEX on craniocerebral injury.

In conclusion, this study demonstrated that DEX protects against post‐TBI inflammatory responses by limiting NF‐*κ*B activation. This effect was likely associated with activation of the Nrf2 signaling pathway. However, future studies are needed to confirm the relationship between Nrf2 signaling and the mechanisms of action of DEX.

## Conflict of Interest

None declared.
